# Supplementary material for: A large scale randomized controlled trial on herding in peer-review discussions
Source: PLoS One. 2023 Jul 12;18(7):e0287443. doi: 10.1371/journal.pone.0287443 (PMC10337975; doi:10.1371/journal.pone.0287443)
Supplement: S1 Appendix — (PDF) [file pone.0287443.s001.pdf]

Appendix

A Caveats regarding the design and analysis of the experiment

Given that our intervention induced the strong difference in the order in which reviewers joined the discussions (see Table 2), the absence of difference in score updates (see Table 3) allows us to conclude with a high degree of confidence that the choice of the discussion-management strategy does not impact the way reviewers update their scores. Of course, herding (if present) does not necessarily need to manifest in how reviewers change their scores after the discussion. Instead, it can change some other characteristics such as what reviewers write in the textual messages which are later analyzed by the area and program chairs who make the final decisions. To account for these potential manifestations, we compared acceptance rates between the groups of papers (see Row 1 of Table 3) and observed some difference in this quantity. However, this difference does not appear significant despite the large sample size we had in the experiment, suggesting that even if present, the effect has at most small size. That being said, we urge the reader to be aware of the following caveats when interpreting the results of this work.

Caveat 1. The design of the intervention. Recall that our research question defines herding as a conditional dependence of the outcome of a paper on the choice of the discussion initiator. The test and the intervention we designed attempt to compare the outcomes of papers when the discussion is initiated by the most positive versus the most negative reviewers with the motivation that this difference is expected to be the largest in the presence of herding. Strictly speaking, the absence of a difference between these choices of the initiators does not imply the absence of the difference between any other choices of the initiators: for example, it is possible that the outcome of a paper would be impacted differently if we asked the reviewer with a non-extreme score to initiate the discussion.

Caveat 2. The choice of papers. As noted in Section 2, in this experiment we tried to identify a set of *borderline* papers as these papers are more susceptible to the impact of the herding effect if it is present. However, our choice of the borderline papers was based on some indirect indicators and hence we could potentially fail to uncover the set of true borderline papers which could reduce the power of our test.

To evaluate our choice of borderline papers, we use a rough classification of submissions into clear and borderline cases made by the area chairs. Note that this classification was performed after the discussion stage which could resolve the uncertainty present before the discussion stage when we selected the participating papers. Hence, the fraction of borderline papers in the area chairs' classification is a conservative estimate of the pre-discussion fraction of borderline papers. Nonetheless, 30% of submissions used in the experiment were classified by the area chairs as borderline cases in contrast to 18% of those not involved in the experiment ($\Delta = 0.12, p = .002$). Hence, our choice of the borderline papers was better than random and the set of the participating papers $\mathcal{P}$ contained a large fraction of papers for which the decisions were not clear before the discussion.

Caveat 3. Satisfaction of Requirement 2 The validity of the conclusions we make is based on the assumption that our treatment scheme satisfied requirements formulated in Section 2. Note that a violation of these requirements not only could increase the false alarm probability, but could also reduce the power of the test. The data we analyzed in Section 3.1 and Section 3.2 strongly supports the satisfaction of Requirements 1 and 2. However, as a note of caution, we remark that there is some

space for potential violations of Requirement 2. Indeed, in Table 1 we establish that the *marginal* values of relevant indicators of discussion activity are similar across groups. However, this observation does not imply that the value of these indicators for each individual paper would not change if that paper was placed in the other condition. Hence, the the outcome of this study should be considered together with this opportunity for the violation of Requirement 2.

Caveat 4. Spurious correlations induced by reviewer identity. In peer review, each reviewer participates in the discussion of multiple papers. Similarly, each area chair manages several papers. Hence, strictly speaking, the outcomes of two papers that have at least one reviewer in common (are managed by the same area chair) may not be statistically independent due to correlations introduced by the reviewer (area chair) identities. Additionally, the limit on the additional burden on reviewers introduced by our experiment (see last subsection of Section 2) makes allocation of papers $\mathcal{P}$ into groups $\mathcal{P}_+$ and $\mathcal{P}_-$ not fully uniform random (some pairs of papers may be required to be placed in the same group to not exceed that limit). These issues put a strain on the testing procedure because in contrast to the vanilla A/B testing framework which assumes that samples are independent of each other, in our case we receive correlated samples. In the domain of empirical studies of the peer-review procedure [3, 18, 46] such spurious correlations are usually tolerated, because otherwise the sample size would be negligible. Additionally, simulations performed by [49] demonstrate that unless reviewers are involved in the discussion of dozens of submissions, the impact of such spurious correlations is limited.

Nevertheless, in this work we take some additional steps to minimize the impact of these spurious correlations. To this end, we simultaneously also perform the analysis on a subset of 937 papers $\mathcal{P}^* = \mathcal{P}_+^* \cup \mathcal{P}_-^*$, where $\mathcal{P}_+^* \subset \mathcal{P}_+$ and $\mathcal{P}_-^* \subset \mathcal{P}_-$ are constructed such that each reviewer is requested to initiate the discussion or contribute to the discussion of at most one paper from $\mathcal{P}^*$ in total. To understand the group construction's significance compare it to $\mathcal{P} = \mathcal{P}_+ \cup \mathcal{P}_-$ which is constructed such that each reviewer is asked to initiate the discussion for at most one paper from $\mathcal{P}$ and contribute to the discussion of at most one paper from $\mathcal{P}$ (at most two requests in total). The additional reduction of the sample size in group construction allows us to limit the impact of the reviewer identity on the outcome of submissions. Of course, by doing so we do not guarantee that there is no reviewer who participates in the discussion of more than one paper from the set $\mathcal{P}^*$, but we guarantee that the discussion participants who are targeted by our treatments are unique. S1 Appendix C gives more details on how sets $\mathcal{P}_-^*$ and $\mathcal{P}_+^*$ were constructed and presents the results of additional analysis on this subset of the papers. Importantly, we note that this additional analysis leads to the same conclusions as in Section 3.

Caveat 5. Opinion of the discussion initiator. In this work we used the scores given by reviewers in the initial reviews to infer the pre-discussion opinion of reviewers and assumed that reviewers begin the discussion from advocating these opinions. However, the fact that a reviewer has some pre-discussion opinion does not guarantee that they advocate the same position in the discussion because the latter is also influenced by other reviewers' reviews and the author feedback. Indeed, past research [17] suggests that reviewers do listen to each other and may update their initial independent opinions in light of opinions expressed in initial reviews of other reviewers. The data we obtained in the experiment suggests that while such updates take place, their magnitude is small enough and does not break our intervention. Indeed, Row 2 of Table 3 and Row 1 of Table 2 indicate that reviewers with extreme pre-discussion opinions remain on the different sides of the mean pre-discussion group opinion (Row 2 of Table 1) even according to the final scores. Thus, we conclude that our intervention succeeded in creating a difference in opinions of discussion initiators across groups.

Caveat 6. Alternative model of herding. In this paper we assume that the herding behaviour in peer review manifests in final decisions being moved towards the position of the reviewer who initiates the discussion. However, the data presented in Table 3 shows that initiators of the discussion tend to slightly update their scores towards the mean of initial scores given by all reviewers. Hence, an alternative model of the herding behaviour is that the sentiment demonstrated by the initiating reviewer carries over to other reviewers who could change their behaviour accordingly. For example, the positive score update of initiators with a negative initial opinion may demonstrate a positive sentiment, which could affect opinions of other reviewers in a positive way. Under this alternative model of herding, we would expect papers from the negative group $\mathcal{P}_-$ to enjoy a higher acceptance rate than their counterparts from the positive group $\mathcal{P}_+$. While this agrees with the observed acceptance rates reported in Table 3, we reiterate that the difference between the acceptance rates is not significant and the effect size is small, so our test does not provide evidence in support of this alternative model either.
