# Supplementary material for: A large scale randomized controlled trial on herding in peer-review discussions
Source: PLoS One. 2023 Jul 12;18(7):e0287443. doi: 10.1371/journal.pone.0287443 (PMC10337975; doi:10.1371/journal.pone.0287443)
Supplement: S2 Appendix — Fig 2. Timeline of the experiment. Day X is the day of the official discussion opening. (PDF) [file pone.0287443.s002.pdf]

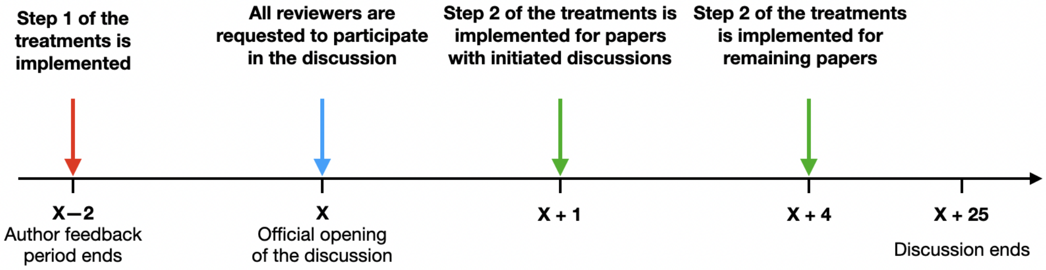

Fig 2. Timeline of the experiment. Day X is the day of the official discussion opening.

B Additional details on the experiment

In this section, we provide additional details on the experimental procedure: timeline of the intervention and selection criteria for papers that were used in the experiment.

Timeline of the Experiment The experiment was conducted over the course of 4 weeks of the ICML 2020 discussion process. Figure 2 depicts the pipeline implemented in the experiment To further increase the power of the experiment, we unofficially opened the discussion portal two days before the scheduled beginning of the discussion and executed Step 1 of both treatments by sending emails to corresponding reviewers. Step 2 of the treatments was then executed in two stages: first, one day after the official opening of the discussion, we executed Step 2 for papers with already initiated discussion. We then waited for three more days before executing Step 2 for all the remaining papers, irrespective of whether the discussion was initiated or not.

Through the first ten days of the experiment, we sent reminders to the reviewers who have not fulfilled our request to initiate or contribute to the discussion of the corresponding papers. In order to avoid a disproportional impact on the discussion participants across two groups of papers, we ensured that the total number of reminders is the same for reviewers who were asked to initiate the discussion and reviewers who were asked to contribute to the discussion.

Construction of the sets $\mathcal{P}_+$ and $\mathcal{P}_-$ We now specify how the set $\mathcal{P} = \mathcal{P}_+ \cup \mathcal{P}_-$ of participating papers (introduced in Section 2) was constructed from the set of all $m = 4,625$ papers not withdrawn from ICML 2020 by the beginning of the discussion period. For this, recall that in Section 2 we mentioned that in order to limit the additional load on reviewers, we require that each reviewer is asked to initiate the discussion for at most one paper from $\mathcal{P}$ and contribute to the discussion of at most one paper from $\mathcal{P}$ (at most two requests in total). To meet this requirement, we construct the target set of papers $\mathcal{P}$ such that each reviewer is the most positive reviewer for at most one paper from $\mathcal{P}$ and the most negative reviewer for at most one paper from $\mathcal{P}$ (the most extreme reviewer for at most two papers from $\mathcal{P}$). To compensate for the associated decrease in the sample size, we design the selection procedure such that $\mathcal{P}$ consists of borderline papers for which the herding effect (if present) is expected to be the most prominent. Having $\mathcal{P}$ constructed, we split it into $\mathcal{P}_+$ and $\mathcal{P}_-$ uniformly at random subject to the aforementioned requirement on the additional burden on reviewers introduced by our intervention. More formally, sets $\mathcal{P}_+$ and $\mathcal{P}_-$ were constructed using the following three-step procedure:

Step 1. First, we identify the set of borderline papers as follows. The overall scores given in the initial reviews were in the set $\{1, 2, \ldots, 6\}$ so for each paper $i \in [m]$, we let λ_i to denote the number of reviewers assigned to the paper (typically λ_i equals 3 or 4)

and let $(\theta_1, \theta_2, \ldots, \theta_{\lambda_i}) \in \{1, 2, \ldots, 6\}^{\lambda_i}$ to denote the collection of overall scores given to paper i in initial reviews. Here, we adopt the standard notation $[\nu] = \{1, 2, \ldots, \nu\}$ for any positive integer ν. With this notation, using acceptance statistics of the ICML 2019 conference, we construct a set of borderline papers $\mathcal{T}$ by identifying submissions that satisfy the following criteria:

C1 The mean overall score is such that in ICML 2019 the paper is in the borderline category:

$$\frac{1}{\lambda_i} \sum_{j=1}^{\lambda_i} \theta_j \in [2.7, 4.5].$$

C2 The minimum and maximum overall scores are on the different sides of the decision spectrum:

$$\max(\theta_1, \theta_2, \ldots, \theta_{\lambda_i}) \geq 4 \quad \text{and} \quad \min(\theta_1, \theta_2, \ldots, \theta_{\lambda_i}) \leq 3.$$

Note that for each borderline paper $i \in \mathcal{T}$ we are guaranteed that there is some disagreement between reviewers.

Step 2. Having the set of borderline papers $\mathcal{T}$ defined, we construct $\mathcal{P}$ by greedily finding a subset of $\mathcal{T}$ that satisfies the requirement of each reviewer being the most positive reviewer for at most one paper from this subset and the most negative reviewer for at most one paper from this subset.

Step 3. Finally, we split $\mathcal{P}$ into $\mathcal{P}_+$ and $\mathcal{P}_-$ uniformly at random subject to the constraint that each reviewer is requested to initiate the discussion of at most one paper and contribute to the discussion of at most one paper (in total, each reviewer receives at most two requests).
