# Supplementary material for: A large scale randomized controlled trial on herding in peer-review discussions
Source: PLoS One. 2023 Jul 12;18(7):e0287443. doi: 10.1371/journal.pone.0287443 (PMC10337975; doi:10.1371/journal.pone.0287443)
Supplement: S3 Appendix — (PDF) [file pone.0287443.s003.pdf]

C Additional analysis

In this section, we report additional analysis that aims to alleviate confounding factors mentioned in Caveat 4 of Appendix 4. Specifically, we replicate the analysis presented in Section 3, conditioning on a subset 937 of papers $\mathcal{P}^* = \mathcal{P}^*_+ \cup \mathcal{P}^*_-$ (see Caveat 4 in S1 Appendix A for motivation and specification of the set $\mathcal{P}^*$).

Construction of the Sets $\mathcal{P}^*_+$ and $\mathcal{P}^*_-$ Recall that $\mathcal{P}^*$ is a subset of the original set of participating papers $\mathcal{P}$ greedily selected such that each reviewer is only approached once by our treatments (that is, is asked to initiate the discussion or contribute to the discussion of at most one paper from $\mathcal{P}^*$ in total). Given that there exist many such subsets of approximately the same size, we facilitate tie-breaking by additionally requesting that for each paper $i \in \mathcal{P}^*$ the most positive and most negative reviewers disagree in the initial reviews by at least 2 points:

$$\max(\theta_1, \theta_2, \ldots, \theta_{\lambda_i}) - \min(\theta_1, \theta_2, \ldots, \theta_{\lambda_i}) \geq 2.$$

Hence, set $\mathcal{P}^*$ has an additional property of containing papers with high disagreement between reviewers in the initial reviews.

Table 4. Comparative statistics on the discussion process

	$\mathcal{P}^*_+$	$\mathcal{P}^*_-$
1. Number of papers	460	477
2. Mean initial score (all revs)	3.51	3.52
3. Standard deviation of initial scores (all revs)	1.22	1.20
4. Mean initial score (revs in discussion)	3.42	3.45
5. Percentage of papers with active discussion	96%	98%
6. Mean number of discussion participants (revs + area chairs)	3.16	3.10
7. Mean discussion length (# messages)	4.52	4.25
8. Percentage of papers with R_+ active in discussion	78%	79%
9. Percentage of papers with R_- active in discussion	87%	85%

Table notes: Comparison of some discussion statistics between two groups of papers ($\mathcal{P}^*_+$ and $\mathcal{P}^*_-$) receiving different treatments. Except Row 4, all values are computed using all papers including those with no discussion. Permutation test at the level 0.05 (two-sided; before multiple-testing adjustment) with 10,000 iterations does not reveal significant differences between conditions in any of the criteria.

Additional analysis on $\mathcal{P}^*$ We now replicate the analysis described in Section 3, conditioning on the set of papers $\mathcal{P}^*$. First, mirroring the analysis on the full set of participating papers (Table 1), Table 4 indicates that various parameters of the discussion are similar across the two conditions even after we condition on the target set of papers $\mathcal{P}^*$. Hence, we also conclude that data supports our treatment scheme in light of Requirement 2 and the intervention did not result in a difference across conditions in the distributions of reviewers who participate in the discussion.

Next, we investigate the efficacy of our intervention and proceed to Table 5 that compares relevant statistics. Observe that the values in Table 5 are very similar to those reported in Table 2, suggesting that the intervention continues to introduce the required difference in opinions of discussion initiators between the groups of papers

Table 5. Does the intervention affect who initiates the discussion?

	$\mathcal{P}^*_+$	$\mathcal{P}^*_-$	Δ	Δ 95% CI	p value
1. Mean initial score (initiator)	4.09	2.69	1.40	$[1.24, 1.55]$	$< .001$
2. Percentage of discussions initiated by R_+	53%	11%	0.42	$[0.36, 0.47]$	$< .001$
3. Percentage of discussions initiated by R_-	16%	60%	-0.44	$[-0.49, -0.38]$	$< .001$

Table notes: The impact of the intervention on who initiates the discussion, conditioned on the subset of papers $\mathcal{P}^*$. To compute values for Row 1, we use 698 papers for which (i) the discussion was initiated, and (ii) the discussion initiator was a reviewer (and not the area chair). For the last two rows, we use all papers including those with no discussion. Bootstrapped confidence intervals are constructed for the difference of the relevant quantities between conditions. All p values for the difference between $\mathcal{P}^*_+$ and $\mathcal{P}^*_-$ are two-sided and computed using the permutation test with 10,000 iterations.

even when we zoom in on the target subset of papers $\mathcal{P}^*$. Thus, we conclude that Requirement 1 remains satisfied and out test continues to posses strong power.

Having confirmed the efficacy of the intervention, we proceed to the comparison of the outcomes of submissions across $\mathcal{P}^*_-$ and $\mathcal{P}^*_+$. The results presented in Table 6 mimic those reported in Table 3, suggesting that conditioning on the set of papers $\mathcal{P}^*$ does not qualitative change the findings. The most notable distinction between Table 6 and Table 3 is that the significance of the difference in acceptance rates (Row 1) becomes closer to the threshold of 0.05 after we condition on $\mathcal{P}^*$, but still does not cross it. Given that the number of submissions involved in the experiment is large, we conclude that we do not observe strong evidence of the herding behaviour even after conditioning on the set of papers $\mathcal{P}^*$.

Table 6. Does the intervention affect the outcome of papers?

	$\mathcal{P}^*_+$	$\mathcal{P}^*_-$	Δ	Δ 95% CI	p value
1. Acceptance rate	0.21	0.26	-0.05	$[-0.11, 0.00]$	.079
2. Change in mean score (initiator)	-0.11	0.21	-0.32	$[-0.42, -0.22]$	$< .001$
3. Change in mean score (all revs)	0.01	0.01	0.00	$[-0.05, 0.05]$	.925
4. Change in mean score (revs in discussion)	0.02	0.02	0.00	$[-0.06, 0.07]$	.867
5. Change in standard deviation of scores (all revs)	-0.26	-0.25	-0.01	$[-0.06, 0.03]$	.560

Table notes: The impact of the intervention on the final outcome of papers, conditioned on the subset of papers $\mathcal{P}^*$. For Row 2, we use 698 papers for which (i) the discussion was initiated, and (ii) the discussion initiator was a reviewer (and not the area chair). For Row 4, we use papers with discussion. For all other rows, we use all papers including those with no discussion. Bootstrapped confidence intervals are constructed for the difference of the relevant quantities between conditions. All p values for the difference between $\mathcal{P}^*_+$ and $\mathcal{P}^*_-$ are two-sided and computed using the permutation test with 10,000 iterations.
