# Supplementary material for: A large scale randomized controlled trial on herding in peer-review discussions
Source: PLoS One. 2023 Jul 12;18(7):e0287443. doi: 10.1371/journal.pone.0287443 (PMC10337975; doi:10.1371/journal.pone.0287443)
Supplement: S4 Appendix — (PDF) [file pone.0287443.s004.pdf]

D Relation to past work on group discussion

Past literature [43–45] suggests the presence of herding behaviour in group discussions. In contrast, in this experiment, we did not detect herding in the peer-review discussion. Let us now discuss the relationship of the current experiment to these past works. First, we note that the papers of [43] and [44] study the herding effect when the discussion initiators are self-selected. The difference between the self-selected and assigned initiators appears to be significant, because the former may be associated with other personal qualities such as assertiveness and energy. Hence, our work is not directly comparable to these studies as we attempt to randomize the identity of the discussion initiator.

The experiment of [45] employs randomization and in addition to the self-selection scenario considers the setup in which the first person to propose the solution to the group is chosen uniformly at random. This work finds that the randomly assigned initiator exerts much smaller influence on the group decision than the self-selected initiator. We caveat however, that in the experiment of [45], the fact that the initiator is selected at random was known to the whole group before the beginning of the discussion. Hence, it is plausible that other group members did not perceive the initiator as a leader and could adjust their behaviour accordingly [45]. In contrast, in the present experiment the non-initiating reviewers were not aware of the intervention and hence from their point of view the assigned initiator of the discussion possessed all the properties of the self-selected initiator [50].

Finally, there is a subtle difference between the definition of the herding effect made by [45] and the definition we use in this paper. According to [45], the herding is present when the first solution formulated in the group discussion predicts the group final decision better than the mean of the pre-discussion independent opinions. Note that according to this definition, the herding may be present even if the first solution proposed to the group is independent of who is selected to formulate this opinion, that is, even when all discussants would propose the same solution should they be selected to start the discussion. In contrast, in our settings it is natural to define herding to be present only when the opinion of the discussion initiator is different depending on who is selected to initiate the discussion, because the goal of the present work is to inform the discussion chairs about the potential consequences of their discussion initiating strategy.

In addition to the aforementioned distinctions from the past work, we note that in the present experiment reviewers are engaged in a much more analytical task as compared to the previous works in which some toy problems were used to study the discussion dynamics. Hence, the absence of the herding behaviour in peer review may be due to the fact that reviewers have a rational mindset which is hypothesized to reduce a reliance on heuristics responsible for various cognitive biases [51, 52].

Beyond testing for herding, in this paper we also document effects predicted by past works on discussion in peer review [17, 21–24]: reviewers tend to update their scores towards the consensus pre-discussion opinion, and the discussion increases the agreement among reviewers. Coupled with the observation made in these past works that an increased agreement does not necessarily result in an increased reliability of the decision, our findings highlight an importance of additional research on the discussion dynamics in peer review.
