# Supplementary material for: A large scale randomized controlled trial on herding in peer-review discussions
Source: PLoS One. 2023 Jul 12;18(7):e0287443. doi: 10.1371/journal.pone.0287443 (PMC10337975; doi:10.1371/journal.pone.0287443)
Supplement: Attachment — Submitted filename: Response to Reviewers.pdf [file pone.0287443.s005.pdf]

RESPONSE TO REVIEWERS

Note from authors: In this document, we respond to questions from the editor and the reviewers. In this letter, we reproduce the text from the editor's and reviewers' responses in **black** and provide our corresponding response in **blue.**

Editor Requirements:

When submitting your revision, we need you to address these additional requirements.

1.Please ensure that your manuscript meets PLOS ONE's style requirements, including those for file naming. The PLOS ONE style templates can be found at

https://journals.plos.org/plosone/s/file?id=wjVg/PLOSOne_formatting_sample_main_body.pdf and

https://journals.plos.org/plosone/s/file?id=ba62/PLOSOne_formatting_sample_title_authors_affiliations.pdf.

Response: We have revised the manuscript to reflect these requirements.

2. Please update your submission to use the PLOS LaTeX template. The template and more information on our requirements for LaTeX submissions can be found at http://journals.plos.org/plosone/s/latex.

Response: We have revised the manuscript to reflect these requirements.

3. Thank you for stating the following in the Acknowledgments Section of your manuscript:

"This work was supported in part by National Science Foundation CAREER award 1942124 and in part by

National Science Foundation Communication and Information Foundations 1763734"

We note that you have provided funding information that is not currently declared in your Funding Statement. However, funding information should not appear in the Acknowledgments section or other areas of your manuscript. We will only publish funding information present in the Funding Statement section of the online submission form.

Please remove any funding-related text from the manuscript and let us know how you would like to update your Funding Statement. Currently, your Funding Statement reads as follows:

"This work was supported in part by National Science Foundation CAREER award 1942124 and in part by National Science Foundation Communication and Information Foundations 1763734.

NSF CAREER award 1942124 was awarded to Nihar Shah
(https://www.nsf.gov/awardsearch/showAward?AWD_ID=1942124&HistoricalAwards=false)

NSF CIF 1763734 was awarded to Nihar Shah
(https://www.nsf.gov/awardsearch/showAward?AWD_ID=1763734&HistoricalAwards=false)

The funders had no role in study design, data collection and analysis, decision to publish, or preparation of the manuscript.”

Please include your amended statements within your cover letter; we will change the online submission form on your behalf.

Response: We do not wish to update the funding statement. We have removed any funding-related text from the revised manuscript.

4. We noted in your submission details that a portion of your manuscript may have been presented or published elsewhere. [DETAILS AS NEEDED] Please clarify whether this [conference proceeding or publication] was peer-reviewed and formally published. If this work was previously peer-reviewed and published, in the cover letter please provide the reason that this work does not constitute dual publication and should be included in the current manuscript.

Response: This work was presented at Peer Review Congress 2022 (https://peerreviewcongress.org/). This venue considers 1-2 page abstracts for submission. The accepted abstracts are not published formally, this is a non-archival venue. Further, the reviewing process is light and considers only the abstract for review.

5. In your Data Availability statement, you have not specified where the minimal data set underlying the results described in your manuscript can be found. PLOS defines a study's minimal data set as the underlying data used to reach the conclusions drawn in the manuscript and any additional data required to replicate the reported study findings in their entirety. All PLOS journals require that the minimal data set be made fully available. For more information about our data policy, please see http://journals.plos.org/plosone/s/data-availability.

Upon re-submitting your revised manuscript, please upload your study’s minimal underlying data set as either Supporting Information files or to a stable, public repository and include the relevant URLs, DOIs, or accession numbers within your revised cover letter. For a list of acceptable repositories, please see http://journals.plos.org/plosone/s/data-availability#loc-recommended-repositories. Any potentially identifying patient information must be fully anonymized.

Important: If there are ethical or legal restrictions to sharing your data publicly, please explain these restrictions in detail. Please see our guidelines for more information on what we consider unacceptable restrictions to publicly sharing data: http://journals.plos.org/plosone/s/data-availability#loc-unacceptable-data-access-restrictions. Note that it is not acceptable for the authors to be the sole named individuals responsible for ensuring data access.

We will update your Data Availability statement to reflect the information you provide in your cover letter.

Response: Data Availability statement: In our work, we analyze data obtained from the scientific conference ICML (International conference on Machine learning) for publication of research in ML. For this, we worked in collaboration with the conference organizers of ICML 2020 (Ivan Stelmakh, Hal Daumé III, Aarti Singh). We are not able to share this data in any form, because of the following reasons:

1. This is highly sensitive data concerning authors and reviewers both, where authors and reviewers provide their honest opinion with the belief that this would not affect them personally.

2. ICML 2020 is a double-blind conference and maintaining the double-blindness is an important aspect of the reviewing process. Releasing de-identified data would dilute the double-blindness.

3. It would violate the confidentiality agreement between the researchers who participated in the conference and the conference organizers.

We are only able to share the aggregated statistics, which are provided in the manuscript.

Further, any data requests may be sent to The International Machine Learning Society that is the institutional body responsible for running the conference ICML 2020, or to the Institutional Review Board of Carnegie Mellon University.

Email ID for CMU IRB: `irb-review@andrew.cmu.edu`

6. Please include your full ethics statement in the 'Methods' section of your manuscript file. In your statement, please include the full name of the IRB or ethics committee who approved or waived your study, as well as whether or not you obtained informed written or verbal consent. If consent was waived for your study, please include this information in your statement as well.

Response: We edited the ethics statement to reflect the requirements of PLOS ONE. It is available on Page 6 of the revised manuscript. We reproduce the edited text here:

"*The deception was approved by Carnegie Mellon University's Institutional Review Board who issued a waiver of informed consent from the participants.*"

7. Please include captions for your Supporting Information files at the end of your manuscript, and update any in-text citations to match accordingly. Please see our Supporting Information guidelines for more information: http://journals.plos.org/plosone/s/supporting-information.

Response: We have revised the manuscript to reflect these requirements.

REVIEWER COMMENTS

Reviewer #1

The paper addresses the topic of the peer review process and how discussions among reviewers might lead to cognitive biases. The authors focus on the peer review process of conferences and grant committees because in these cases a discussion between reviewers is part of the process compared to journals.

Overall, I found the article well written and interesting. I would suggest a few changes to strengthen the manuscript.

My biggest concern with the manuscript is the use of psychological terminologies. In my opinion, the experiment described in the manuscript does not investigate herding behavior but more specifically the possibility of an anchoring effect in the peer review process of conferences. This becomes also clear by the literature that is cited. Tversky and Kahneman, 1974; Strack and Mussweiler, 1997; Mussweiler and Strack, 2001 are all articles investigating the anchoring effect and not herding behavior. I would strongly advice the authors to revise the manuscript to be more accurate with the terminology.

I would also recommend looking further into literature on the primacy and recency effect. Especially with the design of the experiment, the authors should consider that in addition to an anchoring effect there might possibly be a primacy or recency effect. It would also be interesting to examine the length of the discussion although I am not sure if the authors have access to this information.

The recency effect might be a possible explanation for the update towards the mean of the initiators (table 3 row 2).

Although I understand the need for requirement 2 from an experimental perspective, I am not sure if requirement 2 might be the reason why the results do not show evidence for an anchoring effect. Requirement 2 ensured that the discussion is balanced and that the only difference between treatments is the initiators opinion. This might not necessarily reflect the reality of the discussion process. In a discussion where the initiators opinion is dominating, an anchoring effect might occur. The authors should include this fact in the discussion.

A minor comment concerns the explanation of shortcomings of the review system in the introduction. In my opinion the authors should elaborate a bit more to better introduce the aim of their research.

Response: Thank you very much for your suggestions and feedback. We incorporated the suggestions in our manuscript, and the edits are highlighted in blue in the revised manuscript. We further provide our detailed response to the feedback here:

1. In our work we consider the behavior of herding as defined in the seminal paper by Banerjee et. al. [1]. As mentioned in [1], herding behavior may be explained by many psychological internal mechanisms such as anchoring effect, primacy effect, recency

effect. We discuss these possible internal mechanisms in more detail in our revised manuscript. However, it is important to note that our work focuses on observing the outcomes of herding behavior, and not on the internal mechanisms causing the herding behavior. Given the data available, we cannot determine the source of herding behavior, which could be any combination of anchoring, primacy and recency effects, and hence we only focus on the overall outcome of possible herding.

2. Following your suggestion, we provide more discussion about the psychological internal mechanisms that can cause herding behavior in our revised manuscript. We reproduce the text here:

   "*Seminal work by Tversky and Kahneman [30] introduced the anchoring-and-adjustment heuristic, which suggests that after being anchored to an initial hypothesis, humans tend to adjust insufficiently because adjustments are effortful and tend to stop once a plausible estimate is reached. This heuristic is also known as anchoring bias, studied in [31–35]. Another line of work studies the effect of the order of information provision on information retention [36], known as primacy and recency effects. Several human subject studies [37, 38] find that participants remember information better when they appear at the beginning or at the end of a learning episode.*"

   Thank you very much for the suggestion.

3. The review mentions that "it would be interesting to examine the length of the discussion". In our analysis, we examine the length of discussion by measuring the number of separate messages in a paper's discussion thread. This data is provided in Row 7 in Table 1. The text of the individual posts is not available, so we do not measure the length of each individual post in a discussion thread.

Following your suggestion, we provide more information about the shortcomings of the review system in our introduction in the revised manuscript. We reproduce the text here for your reference: *"However, a long line of work identifies various shortcomings of the review system [citations] such as bias in review decisions based on authors' and reviewers' identities, presence of unwanted social influences and human cognitive biases in the decision-making process, and ultimately the lack of consistency in review outcomes."*

Reviewer #2:

The authors carried out a singular study to examine the presence of herding behavior in discussion stage in scientific peer-review. The relevance of this study is justified because of the peer review is a crucial step for publishing research works in the most important scientific

journals. To guarantee that the process is rigorous and is supported in a principled manner, it is necessary to show that the final decision of the peer review is not biased. That is the main goal of this research. For this purpose, the authors carried out an experimental study. The set of papers involved in the experiment was split into 2 groups, balancing the order of positive and negative opinion that initiate the discussion of reviewers. The results showed no evidence of herding behavior in the discussion phase of peer review, but results showed a significant decrease in the variance of final scores due to the agreement among reviewers.

I appreciated having the opportunity to review this manuscript. I think the topic is interesting and relevant in matter of making decisions. However, there are some critical questions and comments that I would like to point out the authors to address, and the final decision should be pending of how the authors are handling the next changes.

Response: Thank you very much for your suggestions and feedback. We incorporated the suggestions in our manuscript, and the edits are highlighted in blue in the revised manuscript. We further provide our detailed response to each comment below.

Major Comments:

1. The introduction section is a little brief. In this section, I miss that the authors summarize deeper the literature of biases making decisions. For example:

a) On paragraph 3 of page 2, the authors wrote: “This finding hints that reviewers within the group reach a consensus not because they identify the “correct” decision, but due to some other artifacts of group discussion” but the authors did not indicate which are these other artifacts of groups discussion, and their implications.

Thank you for pointing that out. We add more discussion about the different artifacts of group discussion in our manuscript. We reproduce the text here:

“*This finding hints that reviewers within the group reach a consensus not because they identify the ``correct'' decision, but due to some other unknown artifacts of group discussion. The work [citation] furthers this investigation, by qualitatively analyzing the group discussions. They find that discourse around score calibration of reviewers plays a pivotal role in the variability in outcomes across different groups. These findings imply that group discussions may lead to a false sense of reliability on a groups' final decision.*”

b) On paragraph 3 of page 2, the authors wrote: “More generally, this observation agrees with a long line of research in psychology (Asch, 1951; Baron et al., 1996; Lorenz et al., 2011; Janis, 1982; Cialdini and Goldstein, 2004) which demonstrates that decisions stemming from a group discussion are susceptible to various biases related to social influence”, but the authors did not indicate which biases related to social influence are talking about and their effects.

Thank you for pointing that out. We add more discussion about the different social influences in group discussions and their effects. We reproduce the text here:

"*More generally, this observation agrees with a long line of research in psychology [citations] which demonstrates that decisions stemming from a group discussion are susceptible to various biases related to social influence, such as confirmation bias, distortion in judgment or compliance due to social pressures based on personal characteristics.*"

c) Also, the authors talk about herding behavior without going into in depth, despite that the research is focus on this bias (see paragraph 5 of page 2).

We add more discussion about the different psychological internal mechanisms behind herding bias in the manuscript. We reproduce the text here:

"*Seminal work by Tversky and Kahneman [citation] introduced the anchoring-and-adjustment heuristic, which suggests that after being anchored to an initial hypothesis, humans tend to adjust insufficiently because adjustments are effortful and tend to stop once a plausible estimate is reached. This heuristic is also known as anchoring bias, studied in [citations]. Another line of work studies the effect of the order of information provision on information retention [citation], known as primacy and recency effects. Several human subject studies [citation] find that participants remember information better when they appear at the beginning or at the end of a learning episode.*"

Further, we discuss the findings from other research on herding behavior in group discussions in more detail. We reproduce the text here:

"*In [citation] it is observed that the first solution proposed to a group predicts the group decision better than an aggregate of initial opinions independently expressed in a pre-discussion survey. The work [citation] documents an impact of the interplay between the status of discussion participants and the opinion of the group member who proposed the first concrete solution on the final group decision. Closest to the present work, [citation] further investigates the herding effect in a semi-randomized controlled trial and declares that the initiators of discussion manage to influence the group opinion when they step in after an initial general discussion of the problem, that is, when they have some understanding of the general opinions of other discussants, but no concrete decisions have been proposed.*"

Hence, I think that the authors should synthesize deeper the literature of biases making decisions (its results and conclusions), especially the herding behavior which is the target of this research.

2. A second critical comment that I would like to point out the authors is regarding to the method section. I think that the explanation of the experimental development is a little confuse and it should be reformulated, being more precise and shorter. It is important that be clear which are the experimental conditions and what each of them implies.

We did not understand this comment by the reviewer. Could the reviewer please elaborate what was imprecise and confusing? Regarding the reviewer's suggestion of being "shorter": we tried to provide the details to be fully rigorous, so we do not understand what the reviewer wishes to condense or eliminate. The experimental conditions and implications are described under the

heading “Treatment scheme” and further discussed under the “Details of the experiment” heading right after that.

3. On the last paragraph of page 4, the authors wrote: “Overall, we designed the intervention with a goal of satisfying the aforementioned requirements. However, a priori we cannot guarantee that these requirements are indeed satisfied in the real experiment”. I think that this paragraph is questioning the reliability and the appropriateness of the experimental design if the authors are not able to guarantee the control of main aspects on their experiment is basing on. Hence, I suggest to the authors remove to the research data those cases that authors cannot guarantee that the experimental requirements are satisfied in the real experiment and repeat the analyses without those data. The authors can report the results with and without these data in order to check their effects.

4. How did the authors handle those cases where the reviewers decided to ignore the discussion phase of peer review, or in those cases where the reviewers that had to initiate the discussion decide no initiate the discussion? Did those cases included in the study data? because I think that those cases no satisfy the requirements…

We combine the responses to comments 3 and 4 since they are aimed at the same issue.

In our analysis, we include the cases where the reviewers decided to ignore the discussion phase or where the reviewer asked to initiate the discussion did not initiate the discussion. The reason for our approach is avoiding selection bias, we explain this in more detail in the next paragraph. Literature in the field of causal inference has a lot of discussion on non-compliance and how ignoring non-complying data points (selecting only complying data points) can lead to biased estimates [1,2]. Our analysis approach is the same as the standard “Intention to treat” analysis commonly applied in causal inference literature [3].

While an analysis on data where the reviewers complied with the experiment design may increase the power of the test, it is very important to note that such an analysis may lead to selection bias and hence violate the false alarm guarantees of the statistical analysis. Specifically, it is possible that the reviewers who comply with the experiment are statistically significantly different in their behavior compared to the reviewers who do not comply with the experiment. If this is the case, then compliance will cause confounding in the analysis that you are suggesting. For instance, consider an example where senior reviewers are more likely to comply with our intervention by initiating the discussion. This could be because senior reviewers are more likely to attend to their reviewing responsibilities. In this example, if we observe herding behavior, it may be explained by the seniority of the discussion initiator if senior reviewers are more likely to be well-calibrated in their reviewing decisions. Hence, the analysis would show herding behavior even though the reason for the effect is not herding.

Finally, the review mentions that these cases (where the reviewer did not comply with the experiment) do not satisfy the requirements of our experiment, however, that is not the correct

interpretation of the requirements posed in the manuscript. The requirements as stated in our work are described for the two groups of papers created in our randomized controlled trial, and not for individual papers. Further, we show that our group-level requirements are satisfied in Table 2.

5. In the section of results, the authors checked firstly whether the requirement 2 was satisfied and then checked whether the requirement 1 was satisfied. As usually researchers answer their research questions in the order in which they are formulated, I think that the authors should check if the requirements are satisfied in the order in which they are proposed in the method section. This is just for a reason of thoroughness and order.

We followed the ordering based on the structure of the narrative of the results section. We consider “requirement 2” first as it is a preliminary requirement and doesn’t indicate the success of our main intervention, whereas “requirement 1” is concerned with the success of our main intervention.

6. I think that the initial and final mean score of initiator, all reviewers, and reviewers in discussion should be reported on Table 3 in addition to the change in those punctuations. That information allows to the readers calculate the change in the punctuations, in order to be more transparent with the data, and to understand better the results. Also, I think that the p value of the changes in those punctuations per every experimental group (P+ and P-) should be reported on Table 3 to check if there were significant differences and, consequently, a herding effect within each experimental group, and not only between experimental groups.

We provide the difference between the initial and final mean scores of (i) initiators, (ii) reviewers and (iii) reviewers that participated in the discussion in Table 3. Further, we provide the initial mean scores of (i) initiators in Row 1 of Table 2, (ii) all reviewers in Row 2 of Table 1 and (iii) reviewers that participated in the discussion in Row 4 of Table 1. This information is provided in this order based on the narrative of the discussion of results. The final mean score for the three groups of reviewers can be straightforwardly deduced from this information.

Further, we provide the p value of the difference across groups in these changes (punctuations) in Rows 2,3 and 4 in Table 3. Our results show that while the difference is significant among the initiators (p value < 0.001), it is not significant for all reviewers or for reviewers that participated in the discussion.

Finally, following your suggestion, we also highlight this in the concluding section. We reproduce the text here:

“*Further, we observe that within each condition, the change in the mean scores of all the reviewers and the set of reviewers that participated in the discussion, before and after the discussion, was small.*”

7. Regarding to the two observations that the authors indicated on results section (see paragraphs 3 and 4 of page 8), any of them actually answer the research question of the paper. In my own opinion, to answer that question, authors should compare the initial and the final

scores of reviewers within each experimental group not between groups. The conclusions that the authors point out are additional conclusions from the results.

Thank you for your suggestion, we updated the discussion section in the revised manuscript to reflect these observations, as mentioned in the previous answer.

Further, we would like to note that in this work, we designed the experiment by having different initiators in the two groups specifically so that the difference in behavior between the two groups will allow us to isolate the effect of herding. Herding within a group, that is the change in initial and final scores within a group towards the initiator, does not necessarily indicate herding behavior. Herding to the initiator within a group could be because of many reasons other than herding. For instance, in our results, we see herding towards the mean in both groups. This implies that if the mean score on average is closer to that of the positive initiator, then we should conclude that there was herding towards the initiator in the group with the positive initiator (P+). However, that is not the case, since this herding behavior is explained by herding towards the mean which is seen in both the groups (P+ and P-).

8. Finally, the discussion section should be extended. I think this section should indicate the main goal of the study, include a brief summary of the main results and conclusions, the theoretical and practical implications of the findings, the main limitations of the study, and some suggestions for future research.

Thank you for this suggestion. We have updated the Discussion section in the revised manuscript to reflect the points mentioned in this comment. We reproduce the updated text here:

"*The results obtained in our work show that \revision{there is no evidence of herding in peer review. While our experiment managed to achieve an imbalance in the opinion of the discussion initiators across conditions, and despite past work having documented an undue influence of the first piece of information on the final decision [citations] in various other settings and applications, the difference in the acceptance rates is not significant and does not suggest the presence of herding behavior in peer review discussions. Regarding policy implications, the absence of the effect suggests that the absence of a unified approach towards discussion management does not result in an increased arbitrariness of the resulting decisions.*" .. "*These caveats present limitations of the study, and are found in the design of the intervention, the choice of papers for the experiment, the satisfaction of Requirement 2, the spurious correlations induced by reviewer identity, opinion of the discussion initiator and the possibility of alternative models for herding.*"

Minor Comments:

9. I suggest clarifying what mean NS, AS and HD (see paragraph 9 of page 5), because it is not reported in the paper.

Thank you for pointing out this lack of clarity in this sentence. We were using these abbreviations to refer to the authors of this paper. We have edited the manuscript to make it clear. NS = Nihar Shah, AS = Aarti Singh, HD = Hal Daumé III.

10. In the section 3.1, the authors wrote: “Finally, we note that most of the papers used in the experiment had some discussion and the length of the discussion is similar across conditions (Rows 5 and 7)”, but the authors did not explain how the length of the discussion was measure. This is important to understand what meaning the length of the discussion (time spent on the discussion, paper extension of the discussion, …).

Thank you for pointing out this lack of clarity about this point. We clarify this in the revised manuscript, text reproduced after comment 11. The length of the discussion for a paper is measured as the number of messages in a paper’s discussion thread.

11. I suggest explaining the row 6 of table 1 in the results section.

Thank you for pointing this out, we have added an explanation of Row 6 in our results section, we reproduce the text here:

“*Finally, we note that most of the papers used in the experiment had some discussion (Row 5). Specifically, we see that the mean number of participants in a paper's discussion and the length of a paper's discussion is similar across the two conditions, where we measure the length as the number of messages in a paper's discussion thread (Rows 6 and 7).*”

References:

[1] E Bareinboim, J Pearl, Controlling Selection Bias in Causal Inference. Proceedings of the Fifteenth International Conference on Artificial Intelligence and Statistics, PMLR 22:100-108, 2012.
[2] M Moerbeek, S van Schie, What are the statistical implications of treatment non-compliance in cluster randomized trials: A simulation study. Journal of Statistics in Medicine. Volume 38, Issue 26, 2019
[3] McCoy CE. Understanding the Intention-to-treat Principle in Randomized Controlled Trials. West J Emerg Med. 2017 Oct;18(6):1075-1078. doi: 10.5811/westjem.2017.8.35985. Epub 2017 Sep 18. PMID: 29085540; PMCID: PMC5654877.
