# Supplementary material for: A large scale randomized controlled trial on herding in peer-review discussions
Source: PLoS One. 2023 Jul 12;18(7):e0287443. doi: 10.1371/journal.pone.0287443 (PMC10337975; doi:10.1371/journal.pone.0287443)
Supplement: Attachment — Submitted filename: Response to reviewers.pdf [file pone.0287443.s006.pdf]

RESPONSE LETTER

Note from authors: In this document, we respond to questions from the editor and the reviewers. In this letter, we reproduce the text from the editor's and reviewers' responses in **black** and provide our corresponding response in **blue.**

Thank you to the Academic Editor and the reviewers for their suggestions and positive feedback about our work! We have incorporated your suggestions in our manuscript. We provide a detailed response to your comments below.

>> AE: In a nutshell, I think the authors have markedly improved the manuscript in the first revision. Reviewer 1 is completely satisfied with your improvements, and regarding the major concerns raised by Reviewer 2, after carefully reading the paper, I detailed which ones also concern me. Thus, consider this report a conditional acceptance once you address my comments in points 3 and 4. If you disagree, please report and explain carefully why you do not follow my advice.

Point 3. I agree with the referee that authors must report, for completeness, the magnitude of the differences between groups.

As suggested by reviewer 2, we have now provided the initial and final mean scores for each group of reviewers in the manuscript. The initial mean scores for different sets of reviewers had been provided in Table 1 (Row 2 and Row 4) and Table 2 (Row 1). In our revision, we provide the final mean scores for each set of reviewers in Section 3.3, while discussing Table 3. We reproduce the text here:

"To understand the effect of the experiment, we first note the observed mean final scores in different sets of reviewers corresponding to groups P+ and P−. Among the reviewers who initiated the discussion, the mean final scores were 4.13 and 2.96 respectively. Among the reviewers who participated in the discussion, the mean final scores were 3.53 and 3.53, and among all reviewers, the mean final scores were 3.47 and 3.48 in P+ and P− respectively."

Further, reviewer 2 pointed out that we should provide the d value:

Reviewer 2: "Also, I suggest to authors indicating the d value (Cohen, 1977) of the differences between groups because this is a statistic that indicates de magnitude of the differences and not only if indeed there are or there are not differences. Reporting the p value is important to know if indeed the differences are statistically significant or are due to random, but we need also know if these differences are small, medium, or large and what is the specific magnitude of the effect."

Following this suggestion, we add the corresponding d value for each row in Table 3 to indicate the magnitude of the effect size.

Point 4. The reluctance of authors to dig deeper regarding the recency and primacy effect is legit. The underpinning of the herding effect is beyond the scope of this paper. However, this point could be included in the discussion as a potential issue to be analyzed by future research.

To address this point in our manuscript, we added the following text to the discussion section: "In this work, we focused on the manifestation of herding effect in peer-review discussions. In future work, it is of interest to understand whether the separate cognitive biases attributed to herding behavior such as anchoring bias [35–39] and, primacy and recency effect [40] have any individual role to play in peer-review discussions."
